# Progesterone Changes the Pregnancy-Induced Adaptation of Cardiomyocyte Kv2.1 Channels via MicroRNA-29b

**DOI:** 10.1155/2022/7145699

**Published:** 2022-04-07

**Authors:** Shuang Liang, Yu-Shuang Sun, Lu Li, Yao Long, Meng Wang, Hou-Zhi Yang, Chun-Di Li, Yan Wang, Shan-Shan Li, Xu Chen, Xin Jin

**Affiliations:** ^1^School of Medicine, Nankai University, Tianjin, China; ^2^Tianjin Central Hospital of Gynecology Obstetrics, Tianjin, China; ^3^Tianjin Key Laboratory of Human Development and Reproductive Regulation, Tianjin, China; ^4^Department of Biopharmaceutical Sciences, College of Pharmacy, Harbin Medical University, Harbin, China; ^5^Tianjin Medical University, Tianjin, China

## Abstract

The cardiovascular system adaptation occurs during pregnancy to ensure adequate maternal circulation. Progesterone (P4) is widely used in hormone therapy to support pregnancy, but little is known about its effects on maternal cardiac function. In this study, we investigated the cardiac repolarization and ion channel expression in pregnant subjects and mice models and studied the effects of P4 administrations on these pregnancy-mediated adaptations. P4 administrations shortened the prolongation of QTC intervals and action potential duration (APD) that occurred during pregnancy, which was mainly attributable to the reduction in the voltage-gated potassium (Kv) current under basal conditions. *In vitro* studies indicated that P4 regulated the Kv2.1 channel in a bidirectional manner. At a low dose (1 *μ*M), P4 induced upregulation of Kv2.1 through P4 receptor, while at a higher dose (5 *μ*M), P4 downregulated Kv2.1 by targeting microRNA-29b (miR-29b). Our data showed that P4 modulated maternal cardiac repolarization by regulating Kv2.1 channel activity during pregnancy. Kv2.1, as well as miR-29b, might be used as potential therapeutic targets for adaptations of the maternal cardiovascular system or evaluation of progesterone medication during pregnancy.

## 1. Introduction

Pregnancy is accompanied by significant changes in the maternal cardiovascular system, which has received much attention in recent years. The maladaptations of the cardiovascular system during pregnancy can lead to severe consequences, such as peripartum cardiomyopathy (PPCM), which is characterized by the decreased cardiac ejection fraction [1–3]. To accommodate the increased metabolic needs and ensure an adequate uteroplacental circulation during pregnancy, the cardiac output increases by 30–50% due to the increased heart rate and stroke volume [1, 2, 4].

Progesterone (P4) is an endogenous steroid, and P4 is involved in the menstrual cycle, pregnancy, and embryogenesis. As a crucial metabolic intermediate, P4 plays an important role in body functions [5, 6]. With good tolerance and few side effects, P4 is currently widely used in hormone therapy to support pregnancy and fertility [7–9]. However, as an agonist of the progesterone receptor (PR), P4 may counteract the action of estrogen in various parts of the body, causing many side effects. During pregnancy, an increase in P4 cooccurs with an increase in heart rate and stroke volume, implicating a pivotal role in regulating pregnancy-induced cardiac dysfunction [5, 10]. Meanwhile, P4 is implicated in regulating action potential duration (APD) and Ca^2+^ uptake in the isolated papillary muscle of rabbit and guinea pig hearts [11, 12]. However, little is known about the molecular events associated with P4 and cardiac dysfunction. The effects of P4 medication during pregnancy are still unclear and need to be further evaluated [13].

Cardiovascular adaptation during pregnancy is associated with the voltage-gated potassium (Kv) channel, which could regulate the strength and duration of heart contraction. Kv channels profoundly affect cardiac repolarization in the form of the QTc interval and/or APD and further regulate the voltage-dependent Ca^2+^ influx (Ca^2+^-dependent release of Ca^2+^ from intracellular stores), allowing the cardiac muscle to more effectively deal with the sudden changes in hemodynamics of pregnancy and parturition [14]. Eghbali et al. have reported that cardiac Kv4.3 channel gene expression is downregulated, accompanied by an increase in the QTc interval and APD prolongation during pregnancy, providing a molecular explanation for cardiovascular adaptations during pregnancy [15]. Therefore, the Kv channel may be a critical factor in the adaptation of the heart during pregnancy. However, the initial factors that led to this process remain poorly understood.

MicroRNAs (miRs), as endogenous, small, noncoding RNAs, have been implicated in various diseases, functioning as posttranscriptional regulators of the expression of multiple genes [16, 17]. Recently, the regulatory role of miRs in human pregnancy-induced cardiac dysfunction has been well established, and hormonally regulated miR dysregulation has also been proven in the endometrium [18]. In particular, miR-29b, a key regulator of Kv channels, is an ideal marker for adequate pregnancy-driven cardiac remodeling [19]. Therefore, in this study, we employed maternal mouse models with P4 administrations and systematically examined P4 effects on Kv channel-mediated cardiac repolarization; the underlying P4-regulated miR-29b mechanisms on the Kv channels were also identified.

## 2. Materials And Methods

### 2.1. Reagents

Antibodies against Kv2.1 (#19963-1-AP, NM_004975) were purchased from Proteintech (Chicago, USA), and an antibody against *β*-actin (#TA-09) was purchased from Zhong Shan Jin Qiao Biotechnology (Shanghai, China). Progesterone (P4, #CSN11782) was purchased from CSNpharm (Shanghai, China). RU486 (#84371-65-3) was purchased from Adooq Bioscience (California, USA). Goat anti-rabbit IgG (7074S, Cell Signaling Technology) and goat anti-mouse IgG (SA00001-1, Proteintech) conjugated with horseradish peroxidase (HRP) were used as secondary antibodies. All other regular chemicals were purchased from standard commercial sources.

### 2.2. Animals and Progesterone Administration

Adult C57BL/6J mice (8-12 weeks old) were purchased from SPF Biotechnology Co., Ltd. (Beijing, China). The animal experiments were approved by the Nankai University's Animal Care and Use Committee (No. 2020KY023). After overnight mating, the pregnant mice were selected and injected with progesterone (P4, 50 mg/kg/day, subcutaneously) from gestational day (GD) 1 to GD 18 (P+P4). The nonpregnant mice (NP) and pregnant mice (P) injected with sesame oil solvent were used for the control groups. At GD 18, the animals were anesthetized with 2.5% isoflurane in oxygen and used for recording electrocardiograms (ECGs, SanRui Medical Device Co., Ltd., China). The duration of each recording was at least 5 min at 25 mm/s with a voltage of 0.5 mV/cm. The hearts were subsequently collected for further studies.

### 2.3. Ethics Statement and Subjects

The study was approved by the Ethics Committee of Tianjin Central Hospital of Obstetrics and Gynecology (2020KY023, April 2, 2020). Pregnant subjects at 37 to 40 weeks of gestation, between 20 and 40 years old, were recruited from Tianjin Central Hospital of Gynecology Obstetrics. In addition, pregnant subjects aged 20–40 years with a clinical diagnosis of threatened miscarriage who received P4 medication (progesterone injection, intramuscular, 10–20 mg daily; oral, 200–300 mg daily) were recruited from Tianjin Central Hospital of Gynecology Obstetrics. The cases involving multiple pregnancies, chronic diseases (e.g., diabetes mellitus and cardiovascular disease), or pregnancy complications (e.g., preeclampsia) were excluded from the study. Pregnant individuals who had received P4 medication for less than one week were also excluded. Age-matched nonpregnant subjects were recruited from the Department of Health in Tianjin Central Hospital of Gynecology Obstetrics as control subjects. Body weight (BW), HR, and blood pressure (BP) were measured just before the study. Finally, 117 pregnant subjects who had received P4 medication, 336 pregnant subjects without P4 medication, and 135 age-matched nonpregnant subjects were recruited at 37 to 40 weeks of gestation for this study. Electrocardiographic recordings of twelve-lead electrocardiograms (ECGs) were obtained using ECG machines (SE-1200 Express, EDAN, China). The QTc intervals were calculated using Bazett's formula (QTc = QT/RR^0.5 and RR = 60/HR). The data were independently analyzed by two investigators and stored on DVDs.

### 2.4. Cardiac Myocyte (CM) Isolation

The mouse CMs were isolated using the simplified, Langendorff-free method [20]. Briefly, the thorax was opened to expose the heart after complete anesthesia. After flushing with EDTA buffer, the heart was sequentially digested with EDTA buffer, perfusion buffer, and collagenase buffer into the left ventricle (LV). The LV was then separated from the other chambers and pulled into 1 mm pieces for CM suspension. The myocytes were enriched in the cell pellets using a 100 *μ*m filter, differential gravity settling, and calcium reintroduction buffers. The cells were collected or resuspended for further studies.

### 2.5. Cell Culture and P4 Exposure

The HEK293 cells (CL-0001; Procell Life Science & Technology Co, Wuhan, China) and the rat cardiomyoblast H9C2 cell line (CL-0089; Procell Life Science & Technology Co, Wuhan, China) were cultured in complete DMEM supplied with fetal bovine serum (FBS) (10%), streptomycin (1%), and penicillin (1%) in a humidified incubator with CO_2_ (5%) at 37°C. The cells of generations two to four were used for experiments. For P4 exposure, we applied 1 *μ*M or 5 *μ*M P4 to mimic endogenous P4 and P4 medication concentrations, respectively. The HEK293 cells and rat H9C2 cells were treated with 1 *μ*M/5 *μ*M P4 with/without 5 *μ*M RU486 (a potent antagonist of the P4 receptor) and cultured for 24-48 h.

### 2.6. Synthesis of miRNAs and Anti-miRNAs

Chemically synthesized and optimized double-stranded nucleotides (m-29b, sense: UUGUACUACACAAAAGUACUG, antisense: GUACUUUUGUGUAGUACAAUU) were designed to mimic endogenous mature miR-29b-5p in cardiac myocytes, and scrambled RNA (NC, CUAAGCCACCAUGUGAAACCAG) was synthesized as a negative control (Sangon Biotech Co., Ltd., Shanghai, China). In addition, one single-stranded antisense oligonucleotide complementary to mature miR-29b (5′-3′: CUAAGCCACCAUGUGAAACCAG) was synthesized to knock down endogenous mature miR-29b in cells.

### 2.7. Cell Transfections and Construction of the Expression Vector

After serum deprivation, the cells were cotransfected with 1 *μ*g of plasmids/oligonucleotides using Lipofectamine (Thermo Scientific, USA) according to the manufacturer's instructions. After 4 h, the cells were switched to complete DMEM with FBS (10%) and cultured for another 24 h. The CDS region of human Kv2.1 mRNA (NM_004975.4) was cloned into pcDNA3.1-CMV-MCS-3xflag-EF1-cherry-T2A-puro (Hanbio Biotechnology Co., Ltd., Shang Hai, China) and expressed in the HEK293 cells. mCherry-labeled fluorescence was used to indicate positive transfections. After 24 h of culture, the cells were used for electrophysiological studies.

### 2.8. Quantitative Real-Time Polymerase Chain Reaction (qRT–PCR) and Western Blot (WB) Analysis

Total RNA of cells/tissue was isolated by TRIzol reagent (Invitrogen, CA, USA) following the manufacturer's instructions. cDNA was then synthesized from total RNA via reverse transcription using the High-Capacity cDNA Reverse Transcription Kit (Thermo Fisher Scientific, MA, USA). Next, qRT–PCR was performed using a PerfectStart Green qPCR SuperMix Kit (TransGen Biotech, Beijing, China) on a SYBR-Green real-time PCR platform (Thermo Fisher Scientific, CA, USA). Specific primers for the Kv channels are presented in Tables 1 and 2. *β*-Actin and U6 was used as the internal controls. The individual result was the average of 3 repeated assays for each experiment. The relative expression of each group was analyzed using the 2^-*ΔΔ*Ct^ method. Proteins were extracted from ventricular myocytes of mouse models and H9C2 cell lines and transferred to polyvinylidene difluoride (PVDF) membranes. After blocking, the membranes were probed with primary antibodies against Kv2.1 (19963-1-AP, NM_004975, Proteintech) and *β*-actin (TA-09, Zhong Shan Jin Qiao Biotechnology, Shanghai, China) followed by incubation with goat anti-rabbit (7074S, Cell Signaling Technology) and mouse (SA00001-1, Proteintech) IgG, horseradish peroxidase- (HRP-) linked secondary antibody, as required. *β*-Actin was used as an internal control.

### 2.9. Electrophysiology

The ruptured*-*patch whole*-*cell configuration of patch-clamp recordings was acquired at room temperature using an EPC10 USB amplifier (HEKA Elektronik) at a sampling rate of 10 kHz. Action potential recordings of isolated myocytes were studied in current*-*clamp mode. The patch pipette electrodes were made from borosilicate glass using a micropipette puller (P-97 model, Sutter Instrument). The resistance of patch pipette electrodes was approximately 3-5 M*Ω*. For action potential recordings, cardiomyocytes were bathed in a solution containing 140 mM NaCl, 5.4 mM KCl, 1.8 mM CaCl_2_, 1.2 mM MgCl_2_, 10 mM HEPES, and 5 mM glucose (pH 7.4). The intracellular solution of the action potential recordings contained 120 mM KCl, 1.5 mM CaCl_2_, 5.5 mM MgCl_2_, 5 mM Na_2_ ATP, and 10 mM HEPES (pH 7.4). Action potentials were elicited by rectangular pulses (2 nA amplitude and 5 ms duration) at a stimulation frequency of 0.5 Hz. For the Kv current recording, the voltage pulse applied was 10 mV voltage steps from -80 to 60 mV with a 500 ms duration, and the holding potential was -80 mV. The extracellular solution used to record the Kv current contained 141 mM NaCl, 4.7 mM KCl, 3.0 mM MgCl_2_.6H_2_O, 1 mM EGTA, 10 mM HEPES, and 10 mM glucose (pH 7.4). The Kv current recordings were performed in the presence of 100 nM iberiotoxin and 10 *μ*M glibenclamide in the extracellular solution. The pipette solution of the Kv current recording contained 125 mM KCl, 4 mM MgCl_2_. 6H_2_O, 10 mM HEPES, 10 mM EGTA, and 5 mM Na_2_ATP (pH 7.4). Single-channel Kv2.1 currents were recorded using the cell-attached method with -80 mV holding potentials. The bath solution in the cell-attached patch-clamp contained 137 mM NaCl, 1.8 mM CaCl2, 5.4 mM KCl, 1.2 mM MgCl_2_, 10 mM glucose, and 10 mM HEPES (pH 7.4). The pipette solution was the same as the bath solution. The average cell capacitance of the H9C2 cells and HEK293 cells were 33.0 ± 8.6 pF (*n* = 29 cells) and 17.2 ± 5.1 (*n* = 26 cells), respectively.

### 2.10. Luciferase Assay

The 432-bp wild-type 3′ untranslated region (UTR) of rat *Kcnb1* (Gene ID: 25736), which contains putative miR-29 binding sites, was synthesized and subcloned downstream of the psiCHECK-2 Renilla luciferase reporter gene (Hanbio Biotechnology, Shanghai, China) (3′-UTR-WT). The sequences from position 7828 to 8259 (counted after the stop codon in the *Kcnb1* mRNA) were cloned into the restriction site of XbaI. The kcnb1-3′-UTR mutated at the 3 residues of 8080, 8082, and 8084 (numbering after the stop codon of the *Kcnb1* mRNA) was subcloned downstream of psiCHECK-2 as a mutant of *Kcnb1*-3′-UTR (3′-UTR-Mut). The 3′-UTR-WT/3′-UTR-Mut was cotransfected with NC/m-29b into HEK293 cells. Following one day of culture, the luciferase activity was measured on a luminometer (GloMax Multidetection System, Promega, USA) using a dual luciferase assay (TransGen Biotech, Beijing, China). The ratio of firefly to Renilla luciferase for each sample was normalized to the control ratio. The cells transfected with NC were used as the negative control.

### 2.11. Immunocytochemistry

The H9C2 cells were grown to 50%–70% confluence in 35-mm Petri dishes and fixed with 4% polyformaldehyde. After blocking with 5% BSA, the H9C2 cells were incubated with an antibody against Kv2.1 overnight at 4°C. On the following day, after washing off the excess antibody with PBS, the H9C2 cells were incubated with secondary goat anti-mouse IgG for 2 h at room temperature. After washing, the nuclei were stained with 4′,6-diamidino-2-phenylindole (DAPI) (A1107, Beyotime, China). The images were captured with a microscope (OLYMPUS, BX51) and analyzed by ImageJ software.

### 2.12. Statistical Analysis

All data are expressed as the mean ± SEM (standard error of the mean). Mean ± SE values were calculated using Excel 2013 software. The significant differences were examined determined using GraphPad Prism 8.0 software (one-way ANOVA followed by the Bonferroni post hoc test or Student's *t* test). When *p* ≤ 0.05, the mean difference was considered significant.

## 3. Results

### 3.1. Cardiac Changes in Electrophysiological Properties during Pregnancy

**Figure 1:**
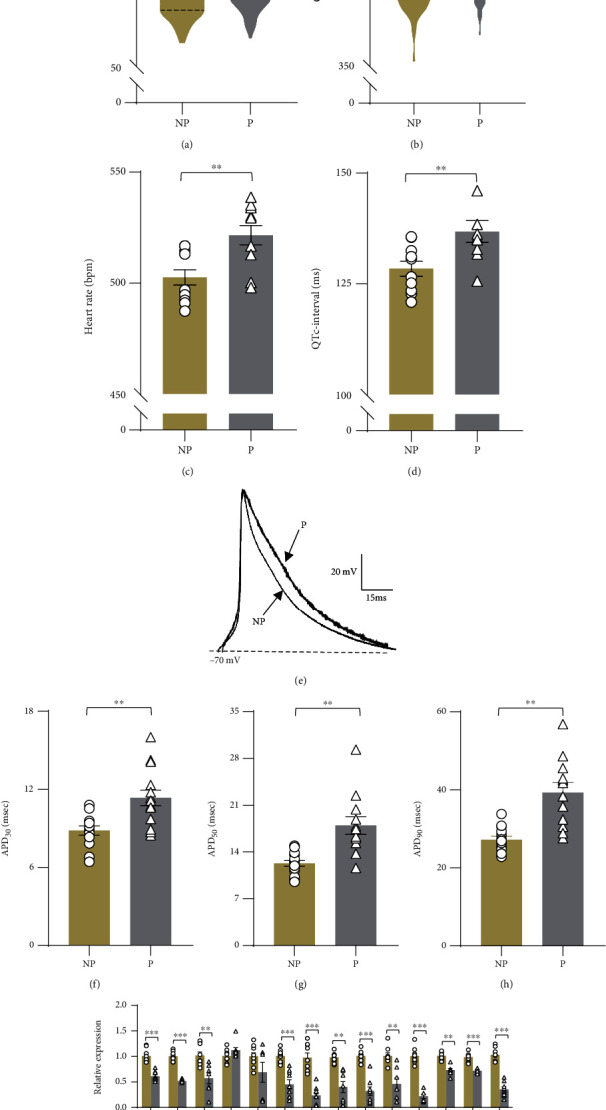
Pregnancy-induced changes in the electrophysiological properties and Kv channel expression of cardiomyocytes. HR (a and c) and QTc interval (b and d) of human subjects (nonpregnant subjects = 135 and pregnant subjects = 336) and model mice (*n* = 9 − 11 animals per group). (e) Representative recordings of the APs in cardiomyocytes stimulated by the rectangular pulse from nonpregnant and pregnant mice. Comparisons of the APD_30_ (f), APD_50_ (g), and APD_90_ (h) between the two groups (*n* = 12 cells from 4 animals per group). (i) qRT–PCR analysis of Kv channels in ventricles from nonpregnant and pregnant mice (*n* = 8 − 9 individual experiments). ^∗∗^*p* < 0.01 and ^∗∗∗^*p* < 0.001 vs. NP. Comparisons of electrophysiological data were conducted by Student's *t* test. Comparisons of qPCR data were conducted by a one-way ANOVA followed by the Bonferroni post hoc test. NP: nonpregnant group; P: pregnant group; HR: heart rate; QTc: corrected QT; APD: action potential duration.

To reveal the cardiac electrophysiological changes in pregnancy, we analyzed the heart rate and heart-rate corrected QT (QTc) intervals of electrocardiographic recordings from 336 healthy pregnant women and 135 age-matched nonpregnant subjects. Pregnant subjects exhibited a significantly higher resting heart rate (HR) than nonpregnant subjects (mean 92.88 ± 1.01 bpm vs. 82.93 ± 1.01 bpm; *p* < 0.01. The heart-rate corrected QT (QTc) intervals were significantly prolonged in pregnant subjects compared with nonpregnant subjects (mean 421.60 ± 0.97 ms vs. 409.91 ± 1.60 ms; *p* < 0.01) (Figure 1(b), Table 3). Increased HR and prolonged QTc intervals were also observed in pregnant model mice, indicating cardiac adaptation with pregnancy status (Figures 1(c) and 1(d)). As the QTc interval reflects repolarization of the myocardial action potential (AP), we next compared cardiomyocyte APs from pregnant and nonpregnant mice. Ventricular myocytes were isolated and recorded with a whole-cell patch clamp (Figure 1(e)). As shown in Figures 1(f) and 1(g), the APD was increased in ventricular cardiomyocytes from pregnant mice (APD_30_: 11.34 ± 0.59 ms; APD_50_: 17.97 ± 1.32 ms; and APD_90_: 40.41 ± 3.07 ms) compared with nonpregnant mice (APD_30_: 8.83 ± 0.35 ms, *p* < 0.01; APD_50_: 12.30 ± 0.42 ms, *p* < 0.01; and APD_90_: 27.20 ± 0.91 ms, *p* < 0.01), indicating prolonged myocardial repolarization during pregnancy.

Voltage-gated K^+^ (Kv) channels play an essential role in the repolarization phase of cardiac AP. Therefore, we used qPCR to detect the effects of P4 on the expression pattern of Kv channel subtypes in the ventricular myocytes from nonpregnant and pregnant mice. As indicated in Figure 1(i), pregnancy caused a decrease in the expression levels of Kv channels in mouse cardiomyocytes, including Kv1.1-1.6, Kv2.1, Kv2.2, Kv3.1, Kv3.2, Kv4.1-4.3, and Kv5.1, indicating a marked downregulation of Kv channel expression during pregnancy.

### 3.2. Effects of P4 Supplementation on Cardiac Action Potentials and Kv Channel Expression during Pregnancy

**Figure 2:**
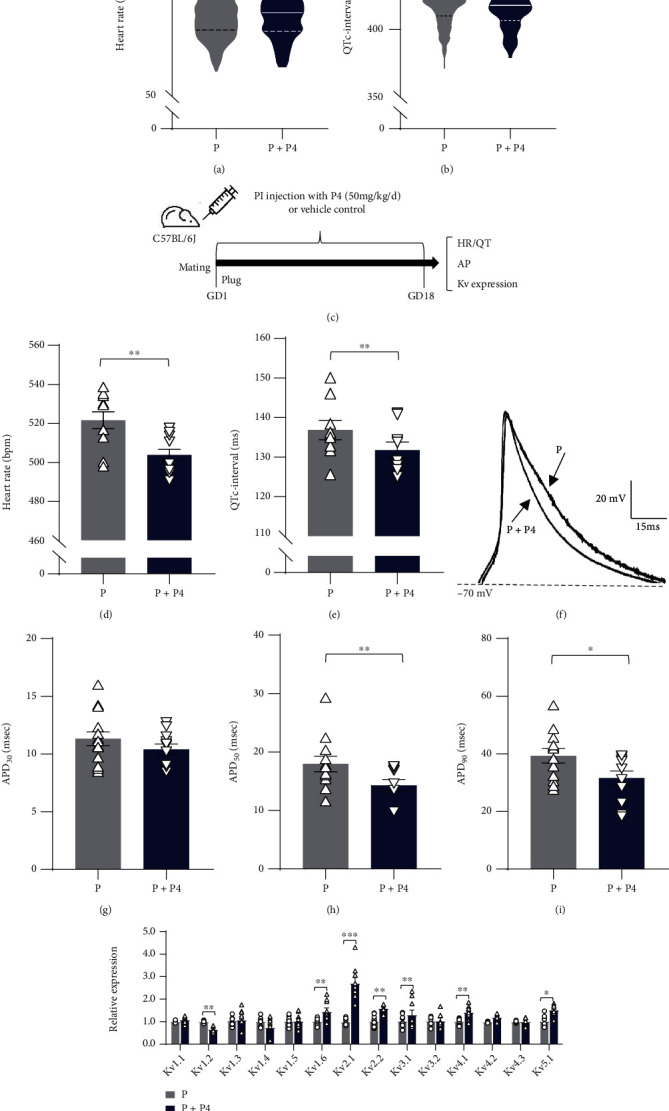
APD and Kv channel expression in response to P4 treatment. Changes in the HR (a) and QTc interval (b) caused by P4 treatment during pregnancy (pregnant subjects = 336 and pregnant subjects who received P4 medications = 117). The pregnant mice were injected daily with P4 (50 mg/kg/d) or sesame oil during gestation for 18 days. Age-matched nonpregnant mice injected with sesame oil served as controls (c). HR (d) and QTc interval (e) of pregnant mice with or without P4 injections. (f) Representative recordings of the APs in cardiomyocytes stimulated by the rectangular pulse from pregnant mice and pregnant mice that received P4 injections. (g–i) Comparisons of APD_30_, APD_50_, and APD_90_ between pregnant mice with and without P4 injections. (j) qRT–PCR analysis of Kv channels in ventricles from pregnant mice and pregnant mice treated with P4. ^∗^*p* < 0.05, ^∗∗^*p* < 0.01, and ^∗∗∗^*p* < 0.001 (*n* = 7 − 10 from 4-6 mice per group). Comparisons of data were conducted by Student's *t* test or one-way ANOVA followed by the Bonferroni post hoc test. P: pregnant group; P4: progesterone; HR: heart rate; QTc: corrected QT; APD: action potential duration.

We next sought to determine whether P4 supplementation could affect the cardiac electrophysiological properties in pregnancy. We compared lead II surface ECG data of pregnant subjects with or without P4 medication. As shown in the ECG parameters in Figure 2(b), P4 medication shortened the QTc intervals in pregnant subjects (417.81 ± 1.51 ms in the P+P4 group vs. 421.60 ± 0.97 ms in the P group; *p* < 0.05). We next investigated the effects of P4 medication on cardiomyocyte APs in pregnant mice. The mice were injected daily with P4 (50 mg/kg/d) or vehicle control (sesame oil) during pregnancy for 18 days (Figure 2(c)). After P4 treatment, ventricular myocytes were isolated and then recorded with the whole-cell patch-clamp technique. Consistent with the findings in human subjects, P4 treatment also affected the QTc intervals during pregnancy in mice (Figures 2(d) and 2(e)). In addition, P4 injection shortened the APD in pregnant mice (APD_30_: 10.79 ± 0.47 ms; APD_50_: 14.31 ± 0.93 ms, *p* < 0.01 and APD_90_: 31.57 ± 2.48 ms, *p* < 0.05) (Figures 2(f)–2(i)).

As indicated in Figure 1, there was a marked inhibition of Kv channel expression during pregnancy. Therefore, we next explored the effects of P4 medication on the expression levels of Kv channel subfamilies in mice during pregnancy. After three weeks of P4 treatment, six Kv channel subtypes were significantly upregulated in pregnant mice, including Kv1.6, Kv2.1, Kv2.2, Kv3.1, Kv4.1, Kv4.2, and Kv2.1, which showed the most apparent modification by P4 medication (Figure 2(j)). These data indicated that P4 medication reversed Kv channel downregulation, especially Kv2.1.

### 3.3. P4 Regulated Kv Channel Activity and Expression in Cardiomyoblast Cells

**Figure 3:**
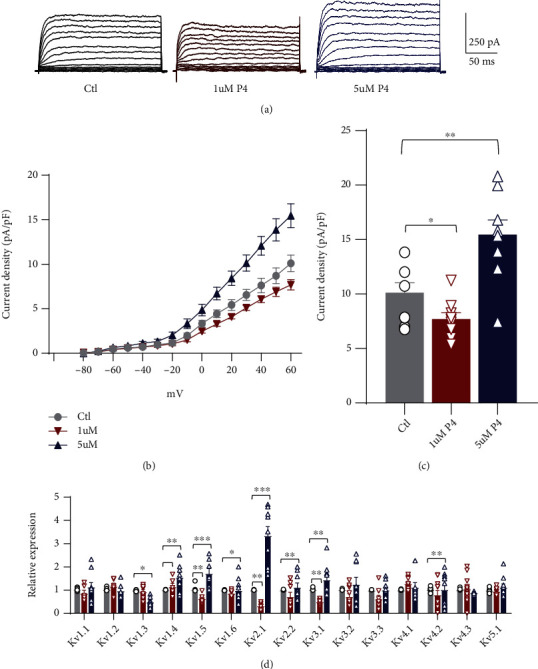
Whole-cell Kv currents and Kv channel expression in response to P4 treatment in the H9C2 myocyte line. The H9C2 cells were exposed to 1 *μ*M or 5 *μ*M P4 and cultured for 48 h. The H9C2 cells without treatment and cultured under normal conditions were used as controls (Ctl). (a) Representative recordings of whole-cell voltage clamp experiments in control, 1 *μ*M P4-, and 5 *μ*M P4-treated H9C2 cells for 48 h. (b) The current–voltage (IV) curve from each group was analyzed at a steady state of the voltage-dependent currents (150 to 180 ms). (c) The current density in each group was compared at +60 mV. ^∗^*p* < 0.05 and ^∗∗∗^*p* < 0.001 vs. Ctl (*n* = 12 − 15 cells per group). Comparisons of data were conducted by a one-way ANOVA followed by the Bonferroni post hoc test. (d) qRT–PCR analysis of Kv channels in the H9C2 cells cultured under 1 *μ*M and 5 *μ*M P4 conditions (*n* = 8 − 9 individual experiments per group). ^∗^*p* < 0.05, ^∗∗^*p* < 0.01, and ^∗∗∗^*p* < 0.001 vs. Ctl. Comparisons of data were conducted by a one-way ANOVA followed by the Bonferroni post hoc test. P4: progesterone.

To further determine the P4 effects on Kv channel activities, we performed electrophysiology studies on the H9C2 cell line. We applied 1 *μ*M and 5 *μ*M P4 to H9C2 cultures to mimic the endogenous concentrations of P4 and additional P4 supplementation during pregnancy. Kv currents were evoked by applying depolarizing pulses between −80 mV and+60 mV from a holding potential of −80 mV (Figure 3(a)). Currents from ATP-sensitive K^+^ (K_ATP_) and Ca^2+^-activated K^+^ (K_Ca_) channels were excluded by adding ATP and EGTA to the intracellular solution and iberiotoxin and glibenclamide to the extracellular solution. No differences were observed in cell capacitance between the control and P4 treatment groups (control: 35.5 ± 8.7 pF; 1 *μ*M P4: 31.9 ± 7.9 pF; and 5 *μ*M P4: 31.7 ± 2.8 pF). Compared with controls, the whole-cell Kv current density in cultured H9C2 cells was suppressed by 1 *μ*M P4 treatment but increased significantly by 5 *μ*M P4 exposure (Figures 3(b) and 3(c)). The current density showed that 1 *μ*M progesterone reduced the Kv current density from 10.1 ± 2.6 pA/pF to 7.7 ± 1.7 pA/pF at +60 mV. In contrast, after 5 *μ*M P4 exposure, the Kv current density at +60 mV increased to 15.5 ± 4.0 pA/pF (Figure 3(c)). Consistently, 1 *μ*M P4 dysregulated Kv channel expression at the mRNA level, including Kv1.5, 2.1, and 3.1 expressions, while 5 *μ*M P4 reversed the effects of low concentrations of P4 on the expression of Kv channels, especially on the expression of Kv2.1 (Figure 3(d)).

**Figure 4:**
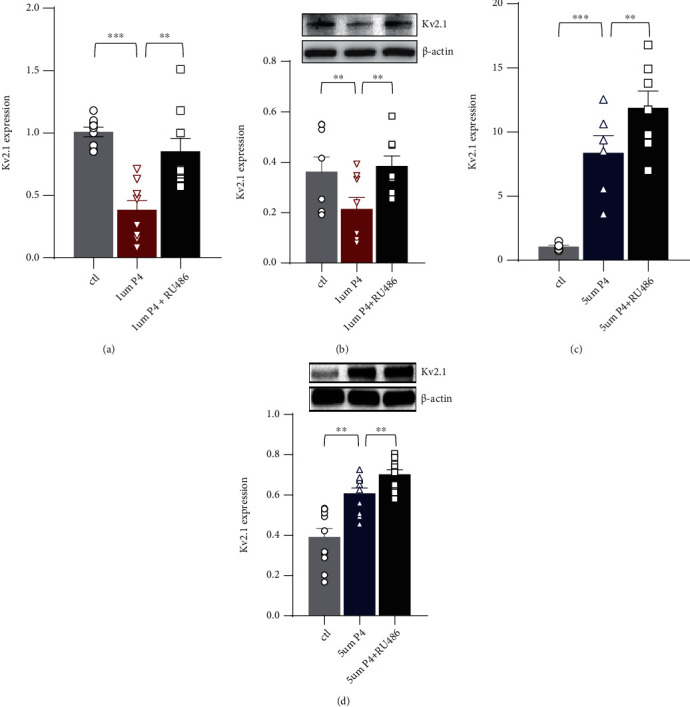
Effects of RU486 on the P4-regulated expression of Kv2.1. The H9C2 cells were exposed to 1 *μ*M P4 and 5 *μ*M P4 and cultured with/without 5 *μ*M RU486 for 48 h. The H9C2 cells without any treatment and cultured under standard conditions were used as controls (Ctl). (a and c) qRT–PCR analysis of Kv2.1 in the H9C2 cells cultured under 1 *μ*M and 5 *μ*M P4 conditions with/without 5 *μ*M RU486 application. (b and d) WB analysis of Kv2.1 in the H9C2 cells cultured under 1 *μ*M and 5 *μ*M P4 conditions with/without 5 *μ*M RU486 application. ^∗∗^*p* < 0.01 and ^∗∗∗^*p* < 0.001 vs. Ctl (*n* = 4 − 6 individual experiments). Comparisons of data were conducted by a one-way ANOVA followed by the Bonferroni post hoc. P4: progesterone; RU486: an antagonist of P4; qRT–PCR: quantitative real-time polymerase chain reaction; WB: western blot.

In the 5 *μ*M P4 group, the mRNA expression levels of Kv2.1 in the H9C2 cells were approximately fivefold higher than those in the controls. In addition, the effect of 1 *μ*M P4 on Kv2.1 could be reversed by administering 5 *μ*M RU486 (Figures 4(a) and 4(b)), which is a potent antagonist of the P4 receptor. In contrast, the effect of 5 *μ*M P4 on Kv2.1 was not affected by RU486 (Figures 4(c) and 4(d)). These results indicated that P4 medication reversed the pregnancy-related inhibition of Kv2.1 expression through a new mechanism other than the classic receptor-binding pathway.

### 3.4. P4 Facilitated Kv2.1 Channel Expression through MicroRNA-29b (miR-29b)

**Figure 5:**
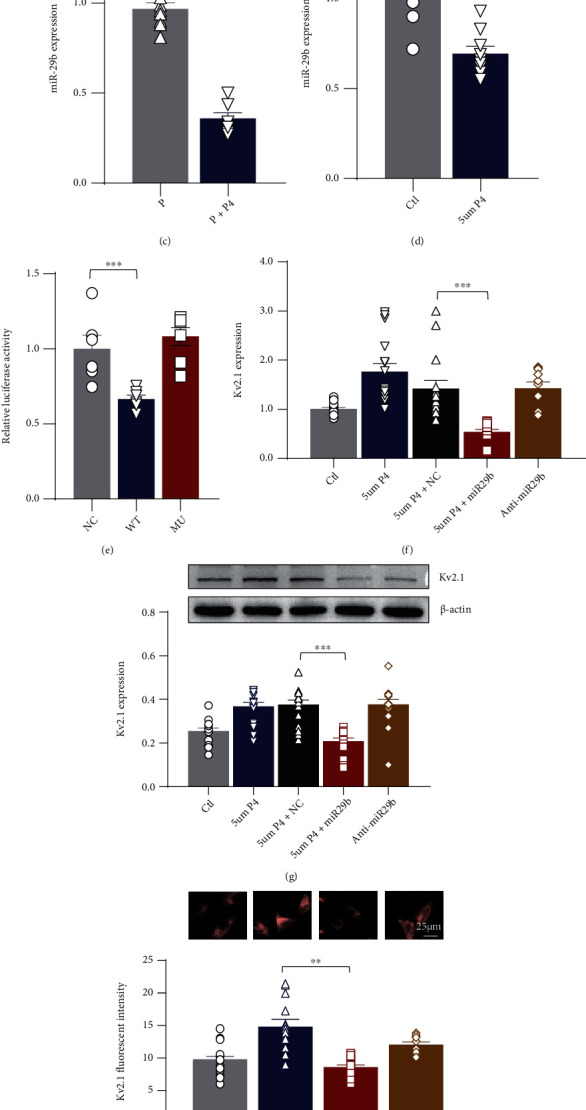
Validation of Kv2.1 miR-29b targeting. (a) Schematic illustration of the hypothesis that progesterone facilitated Kv2.1 channel expression through miR-29b. (b) Structure diagram of the luciferase reporter of kcnb1 and its mutant. Seed match regions of kcnb1 and miR-29b are indicated as vertical lines. The mutation site of kcnb1 mRNA is indicated in blue. (c) qRT–PCR analysis of miR-29b in the ventricles of pregnant mice with/without P4 injections. (d) qRT–PCR analysis of miR-29b in the H9C2 cells treated with 5 *μ*M P4 for 48 h. The H9C2 cells cultured without treatment served as negative controls (Ctl). (e) Luciferase assay for kcnb1. miR-29b mimics were cotransfected with the wild-type 3′-UTR of kcnb1 (kcnb1-WT) or its mutant (kcnb1-M) into 293 cells. Luciferase activities were measured after 24 h of culture (*n* = 3 individual experiments). qPCR (f), WB (g), and immunocytochemistry analysis (h) of Kv2.1 expression were conducted in the H9C2 cells from the different transfection groups that were cultured under 5 *μ*M P4 conditions. The H9C2 cells transfected with scrambled RNA and cultured without P4 exposure were used as controls (NC). ^∗∗^*p* < 0.01 and ^##^*p* < 0.01 (*n* = 4–6 individual experiments; at least 6–10 fields from each group were imaged and scored in a blinded fashion). Comparisons of H9C2 data were conducted by a one-way ANOVA followed by the Bonferroni post hoc or Student's *t* test. P4: progesterone; qRT–PCR: quantitative real-time polymerase chain reaction; WB: western blot.

As the emerging role of microRNAs in a variety of physiological and pathological processes, we hypothesized one potential mechanism for the alteration of Kv2.1 stability was through miRNAs targeting. Therefore, we looked into the online database for miRNA-mRNA prediction (http://mirdb.org/) and found that microRNA-29b (miR-29b) was in high ranking for Kcnb1 prediction scores (http://mirdb.org/cgi-bin/target_detail.cgi?targetID=2290938). The bioinformatics predictions showed that rat *Kcnb1* was a potential target gene of miR-29b with seven complementary binding sites located in the 3′-UTR of the *Kcnb1* gene (Figure 5(b)). miR-29b is highly conserved across species and expressed in the heart. Maternal cardiac changes in miR-29b are tissue-specific and related to maternal obesity in baboons [21]. Additionally, miR-29b is involved in pregnancy regulation, regulating the deceleration of postnatal body growth [21]. Recent studies have confirmed that the miR-29b family is involved in cardiac remodeling during pregnancy, regulating fibrosis and cardiac hypertrophy by targeting mRNA [21–24]. Based on the literature, we specifically focused on miR-29b to explore whether miR-29b was involved in P4-regulated Kv2.1 activity (Figure 5(a)).

We studied the effects of P4 medication on the expression of miR-29b in cardiomyocytes from maternal mice and found that P4 injections inhibited the expression of miR-29b during pregnancy, which was further proven in the H9C2 cells treated with 5 *μ*M P4 (Figures 5(c) and 5(d)). Next, we constructed several constructs to demonstrate the potential interactions between miR-29b and Kv2.1 (Figure 5(b)). In luciferase assays, miR-29b significantly reduced wild-type *Kcnb1* (kcnb1-WT) luciferase activity, whereas this effect was abrogated when the miR-29b binding sites at 8080 in the 3′-UTR of *Kcnb1* were mutated (Figure 5(e)). To further understand whether miR-29b mediated the P4 regulation of Kv2.1, we examined the expression of Kv2.1 in the H9C2 cells when miR-29b was either overexpressed or antagonized under 5 *μ*M P4 exposure and found that there was an inverse correlation between miR-29b and Kv2.1 expression. The expression of Kv2.1 was significantly enhanced by 5 *μ*M P4 exposure in the H9C2 cells, and this increase was efficiently prevented by miR-29b overexpression, as confirmed by qPCR (Figure 5(f)), western blot (WB) (Figure 5(g)), and immunocytochemistry analyses (Figure 5(h)).

### 3.5. Regulation of Human Kv2.1 Channel Activity by P4

**Figure 6:**
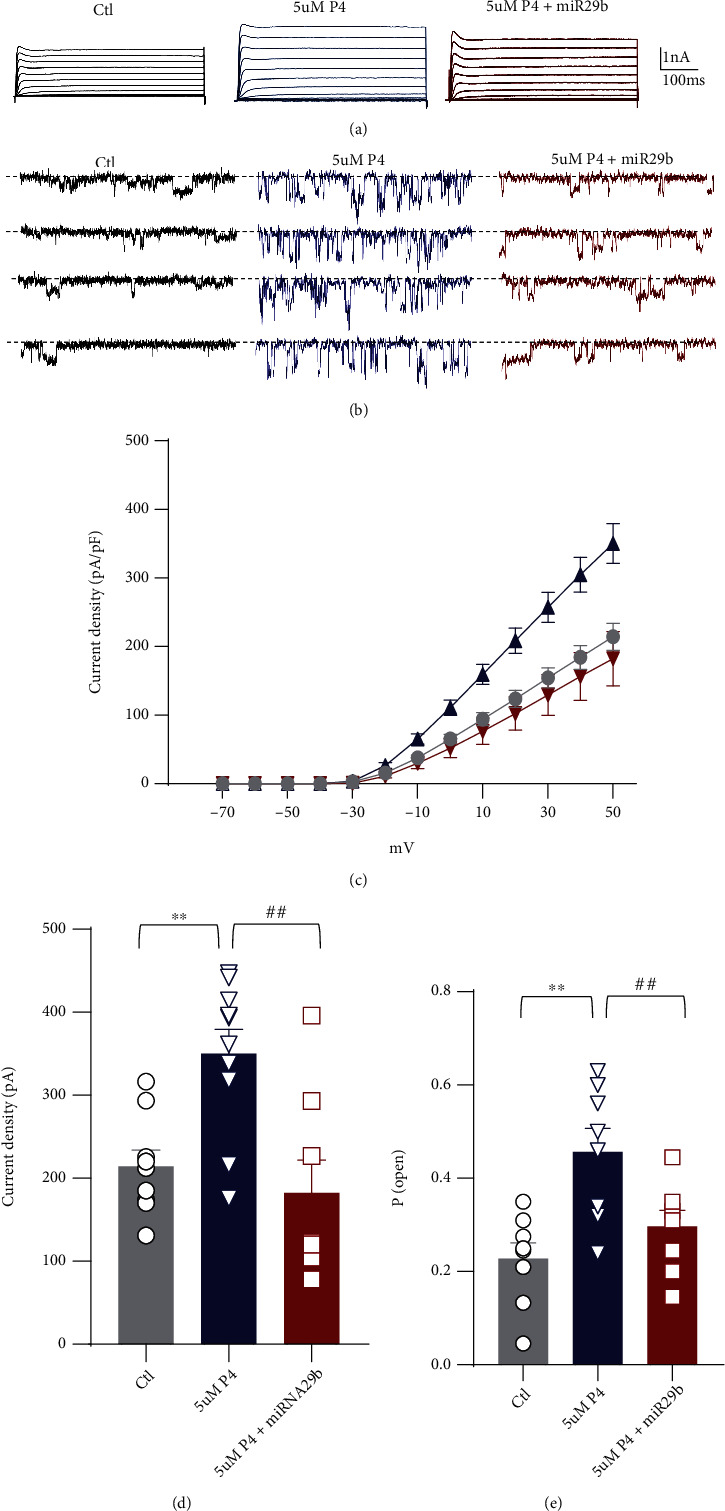
Effects of P4 on human Kv2.1 channel activities. (a) Representative Kv2.1 current traces derived from control, 5 *μ*M P4 treatment, and 5 *μ*M P4 with miR-29b mimics. (b) Representative Kv2.1 single-channel traces derived from control, 5 *μ*M P4, and 5 *μ*M P4 with miR-29b mimics. (c) Current–voltage (I–V) relationships of the steady-state Kv2.1 currents. (d) The current density at +50 mV with control, 5 *μ*M P4, and 5 *μ*M P4/miR-29b. (e) Changes in the probability of Kv2.1 channel opening. Values shown are the mean ± S.E.M. ^∗^*p* < 0.05 and ^∗∗^*p* < 0.01 vs. Ctl; ^#^*p* < 0.05 and ^##^*p* < 0.01 vs. 5 *μ*M P4 (*n* = 9 − 10 cells per group). Data were compared by a one-way ANOVA followed by the Bonferroni post hoc test. P4: progesterone.

We further characterized whether P4 has a similar effect on human Kv2.1 channel activity. Patch-clamp recording was performed in human embryonic kidney 293 (HEK293) cells expressing human Kv2.1 with or without 5 *μ*M P4 treatment. From a holding potential of −80 mV, the Kv currents were evoked by applying depolarizing pulses between −80 mV and+60 mV. No significant differences in cell capacitance of HEK293 cells were found among all groups (control: 18.2 ± 5.8 pF; 5 *μ*M P4: 16.1 ± 4.9 pF; and 5 *μ*M P4+miR29b: 17.8 ± 4.8  pF). However, compared to the control, exposure to 5 *μ*M P4 for 48 h markedly increased the Kv2.1 outward current (Figure 6(a)). At +50 mV, the Kv2.1 current density increased to 350.1 ± 91.7 pA/pF in the 5 *μ*M P4 group, which was approximately 30% higher than that in the control group (214.2 ± 59.4 pA/pF). This increase in the Kv2.1 current with 5 *μ*M P4 was eliminated by miR-29b transfection (Figures 6(c) and 6(d)). We next examined the effects of P4 on Kv2.1 single-channel properties (Figure 6(b)). As shown in Figure 6(e), the single-channel recording showed that 5 *μ*M P4 treatment significantly increased the open probability of the Kv2.1 channel in HEK293 cells, and this effect was suppressed in the presence of miR-29b. These findings suggested that the augmented effects of 5 *μ*M P4 on Kv2.1 channel activity were likely due to inhibition of miR-29b.

## 4. Discussion

P4, known as the “pregnancy hormone,” supports implantation and pregnancy, widely used as a hormone supplement during pregnancy. However, the increase in P4 cooccurs as the increase in heart rate and stroke volume, indicating adaptations of the cardiovascular system during pregnancy. Little is known about the effects of P4 administrations on cardiac adaptions during pregnancy. Herein, we provided evidence that P4 bidirectionally regulates Kv2.1 channel activities, providing new evaluations for P4 administrations during pregnancy.

**Figure 7:**
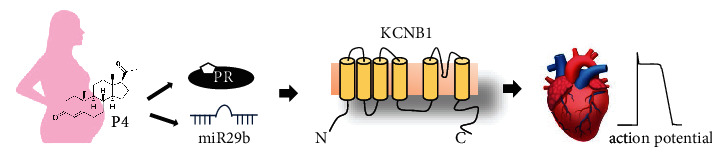
Schematic illustration summarizing the conclusion that progesterone regulated cardiomyocyte Kv2.1 channels via microRNA-29b and progesterone receptors.

The result showed that the myocardial repolarization was prolonged in pregnant women and mice and P4 bidirectionally regulated myocardial repolarization and Kv 2.1 functions. Notably, P4 positive regulated Kv2.1 expression through the PRA/B pathway, but negative regulated Kv2.1 expression through the miR-29b pathway under different doses of P4 (Figure 7). This bidirectional regulatory mechanism of P4 on cardiac adaptation during pregnancy, especially the negative regulation through miR-29b, implied that excessive P4 abundance or increased sensitivity might be a potential risk factor for cardiac maladaptation. To the best of our knowledge, the present study is the first to demonstrate that miR-29b is regulated by P4 and is involved in Kv2.1 channel regulation, which is a possible mechanism for cardiac maladaptation. Furthermore, miR-29b could be a biomarker to predict as well as a drug to cure maladaptation of the cardiovascular system.

Cardiac adaptation in pregnancy cannot be researched by a direct method, but cardiac repolarization is an excellent indirect indicator used to evaluate the peripartum myocardial burden in pregnant women [25]. In mammalian ventricles, repolarization can strongly regulate cardiac contractility due to its adjustment in absolute and relative refractory periods as well as the calcium concentration in myocardial cells [26–29]. Compared with nonpregnant control subjects, prolonged repolarization was observed in the first trimester and continued into the late pregnancy and the postpartum period with the increased cardiac output [14, 30, 31]. Our results showed that the QTc interval was prolonged in pregnant women and mice. At the same time, the cardiac APD of pregnant mice was also prolonged. Furthermore, PCR analysis showed a remarkable alteration in the expression patterns of Kv channels, and nearly twelve Kv channels were downregulated in pregnant mice.

The increased APD and prolonged QTc are the main cardiac adaptations during pregnancy, which are combined with the increased level of endogenous P4. When pregnancy occurs, P4 levels may increase to 1 *μ*M at term [32]. However, women who at risk for threatened abortion or *in vitro* fertilization (IVF) pregnant women typically need additional P4 support (20 mg intramuscular injection), which is associated with a higher incidence of cardiovascular disease [33–35]. Therefore, we investigate the P4 effect with low/high doses *in vivo* and *in vitro* to show the implications for the additional P4 support during pregnancy. During the late phase of pregnancy, the P4 level in fetal circulation (4.5 *μ*M) is much higher than that in maternal blood (1 *μ*M), which may be the reason for the high rate of sudden death in the uterus [32].

Additionally, a previous study reported that 5 *μ*M P4 inhibited the current density and protein trafficking of a HERG channel [36]. In the present study, we applied 5 *μ*M as a high dose of P4 to study its effect on Kv channels. The results showed that 1 *μ*M and 5 *μ*M P4 had a bidirectional effect on Kv2.1 activity, decreasing and increasing Kv2.1 current density, respectively. Our study further indicated that the difference in P4 effects on different ion channels might involve various mechanisms/pathways. We also tried to test a higher dose of P4 ranging from 10 to 20 *μ*M on the H9C2 cell line, whereas it produced cytotoxicity (data not shown). We found that the regulation of cardiac repolarization during pregnancy by P4 was bidirectional. In previous studies, P4 has been documented to shorten cardiac repolarization [11, 37, 38]. Our study found that P4 bidirectionally regulates APD in pregnant mice, and we further confirmed our findings by checking the expression of Kv channels *in vivo* and *in vitro*. Myocardial repolarization during pregnancy was prolonged in humans and mice, and the Kv channel, which governed myocardial repolarization, was regulated by P4 *in vitro*.

However, our study was not enough to evaluate the clinical P4 treatment during pregnancy. The P4 produced a bidirectional effect on the cardiac system which may be viewed as a hormetic phenomenon of female sex hormones [39], like dose-related neuroprotective versus neurodamaging effects of progestagens in animal stroke models, which was mainly caused by a hormetic phenomenon due to underestimating the importance of well-established administration regimens [40–43]. Our results showed that the P4 produced bidirectional responses on cells/animals which may occur in multiple mechanisms (like miR-29b, receptors, and hormetic effect) with different potency and efficiency taken together, implying the importance of well-established P4 administrations. Therefore, the bidirectional actions of P4 on Kv2.1 reflected the combined effects of P4 on receptors and miRs, which had important implications for the clinical phenomenon of cardiac adaptation in normal pregnancy and the high incidence of cardiovascular disease in *IVF* pregnant women requiring additional P4 support.

The regulation of the Kv2.1 channel by P4 was concentration-dependent and occurred via different pathways. Kv2.1 was reported to play conductive and structural roles in regulating membrane potential, intracellular Ca^2+^, and the myogenic tone of arterial myocytes [44]. Cattaneo et al. reported that the expression of Kv2.1 may be regulated by sex hormones [45]. We found that the different doses of P4 produced opposite effects on the expression of Kv2.1 in the H9C2 cell culture. That is, there was upregulation at a low dose (1 *μ*M) via progesterone receptor (PR) and downregulation at a high dose (5 *μ*M) via miR-29b. It was suggested that the Kv2.1 channel might be a key factor in P4-induced cardiac adaptation through different pathways.

miR-29b is an important regulatory factor in the adaptation of the heart during pregnancy. Recent studies have confirmed that the miR-29b family is involved in cardiac remodeling during pregnancy, regulating fibrosis and cardiac hypertrophy by targeting mRNA [21–24]. Our results showed that Kv2.1 was a direct target of miR-29b, and miR-29b rescued the effects of 5 *μ*M P4 on Kv2.1 expression. Electrophysiology studies further confirmed that miR-29b mediated P4 actions on Kv2.1 channel currents. To the best of our knowledge, the present study is the first to demonstrate that miR-29b is regulated by P4 and is involved in Kv channel regulation. Meaningfully, dysregulation of miR-29b may reflect the effects of P4 on cardiac adaptation, indicating that it is a novel biomarker for predicting maladaptation of the cardiovascular system during pregnancy.

There are still limitations in the present study. First, the study was limited to animal models, which were different from humans in terms of an electrical matrix, cell current, channel dynamics, cardiac AP and electrocardiogram, pregnancy-related timing, and endogenous hormone changes. Still, the pregnant mouse model we used was the best existing model to study electrophysiological changes in the heart during pregnancy, as these animals exhibited increased heart rate and prolonged QTc intervals similar to those in human pregnancy [9]. Second, the pregnant mice results cannot be considered a fully appropriate extrapolation for human subjects because of the differences in Kv2.1 channel subunit expression, subunit composition, and function. Kv2.1 is a slow delayed-rectifier K^+^ channel that conducts the slow K^+^ current in repolarization in rodent ventricular myocytes [46–48]. Although Kv2.1 RNA and protein have been detected in human atrial myocytes and ventricles, studies are still needed to investigate further their role in regulating human cardiac repolarization [49, 50]. Third, during P4 administration, other hormones' influence was not excluded. As hormones are necessary to maintain pregnancy, it is impossible to use hormone receptor antagonists or knockout mice to eliminate their effects during pregnancy. Therefore, pregnant mouse models injected with P4 were compared with normal pregnant mice to ensure the same production of other hormones during pregnancy. Last, this study did not collect data on the repolarization, P4, and miR-29b levels of PPCM patients due to the low incidence of the disease. Furthermore, RU486 is not specific to progesterone receptors but also inhibits glucocorticoid receptor pathways. In the absence of progesterone receptor-specific antagonists, the use of RU486 on P4-treated H9C2 cell lines still ruled out the involvement of intracellular hormone receptors (progesterone and glucocorticoids), suggesting a novel mechanism by which P4 regulates Kv2.1 expression (mir-29b). Of course, specific progesterone receptors knockout/antagonist would be more convincing for mechanism studies. Further studies will be carried out in the near future.

## 5. Conclusion

In conclusion, P4 bidirectionally regulated Kv channels involved in cardiac adaptation during pregnancy and negatively regulated Kv2.1 activity through miR-29b. These findings revealed the mechanism of normal cardiac adaptation during pregnancy as well as maladaptation in gestational disorders, such as PPCM. Kv2.1, as well as miR-29b, might be helpful for the evaluation of P4 medication during pregnancy and considered as a potential therapeutic molecule to correct the maladaptations of the maternal cardiovascular system.

## Figures and Tables

**Table 1:** qRT-PCR primers for the HEK293 cells Kv channel mRNA and actin.

mRNA	Forward primers (5′-3′)	Reverse primers (5′-3′)
Kv1.1	TCCGTCATGGTCATCCTCATCTCC	GGTGAATGGTGCCCGTGAAGTC
Kv1.2	CATTTTGTACTACTACCAGTCTGGGG	GGAGTGTCTGGACAACTTGAAAATCC
Kv1.3	AGCTTCGACGCCATCCTCTACTAC	TCCATAGCCTCCTCACCCAGTTG
Kv1.4	TCTCTTACAACTGGAACCAGC	TGAGGATCATGGGAGGAGT
Kv1.5	GAGGTTTTTCCCGCTGCCTGG	TCCAGGGGCACCAGGGAGAT
Kv1.6	AGAGACGGAGCAGGAGGAACAAG	AGTCAGGTTTGCCAAGCCCATTG
Kv2.1	CAATCTGTGGACACTAACTAGC	GCAGAAAGAATTGAGTCTAAATTGT
Kv2.2	ACTGACTTCACGGAGACTGAGAGG	CTGGGAGGTCGGTGGGGAAC
Kv3.1	CTCTTCTTCATCCTGGTCTC	CCAGGAAGATGATAAGCAGC
Kv3.2	ACTTGGTGCTGTGTTCGCCTTC	GCCTGGTGCCTCCGACATTG
Kv3.3	TCTTCGCCTACGTGCTCAAC	CGCGTCCTGAAAACACAGAC
Kv4.1	TGGACCGAACATCTACCGACTCTG	ACAGCACCCATCTACCCATCTACC
Kv4.2	CCGTGCCTGTGATCGTGTCTAAC	CCTCGCTTTCTTCTGTGCCCTTC
Kv4.3	CCTCCGCCAGCAAGTTCACAAG	TGACCAGGACGCCGCTTAGG
Kv5.1	TTGGACGACCTGGGTGTAGACG	ACCACGGAGGAGACAAGGATGAG
U6	GCTTCGGCAGCACATATACTAAAAT	CGCTTCACGAATTTGCGTGTCAT
rno-miR-29b-5p	GCGCTGGTTTCACATGGTG	AGTGCAGGGTCCGAGGTATT

**Table 2:** qRT-PCR primers for mouse Kv channel mRNA and actin.

mRNA	Forward primers (5′-3′)	Reverse primers (5′-3′)
Kv1.1	TCAGTTGCTCCATGTTAGTTCT	CTGTCTGTAATGGGCTATGCTA
Kv1.2	TCGAAACTCAGCTAAAGACCTT	CTCCTAGCTCATAAAACCGGAT
Kv1.3	ATCCTCTACTACTACCAGTCCG	CCTCATCCTCACGGAACTTTTC
Kv1.4	ATATGCCTTATGGTTATGCAGC	GTTTCTTCTTCTCAGTTCGCTG
Kv1.5	CACTATGACCACTGTAGGCTAC	GTCTGTCTCCCGATGATAGAAG
Kv1.6	GTCTGTCTCCCGATGATAGAAG	CAACTGATAGAAGCGGATCTCT
Kv2.1	GGCTTGTATCACGATCCTCTTA	CATCAGTGTCGGTGTCTATGTA
Kv2.2	CCAATTGACATAACCGTGAACC	GGCTCTGTTTTCCTGAAAGTTC
Kv3.1	ACAGCCACTTCGACTATGAC	AATAGTTCAGGATGTGAGCGAA
Kv3.2	GCAAGATCGAGAGCAACGAGAGG	TGAGGTTCAGAGGAGGCAAGAAGG
Kv3.3	CTGCTGCTGGATGACCTATC	CTGAAAACACAGACGCTTGAG
Kv4.1	GTGCTGAACTTTTATCGCACTG	TGTACTCTTCAAGACAGCAGTC
Kv4.2	GAACTTCAGTCGGATCTACCAC	GCTGGATCCAGATTTGCTTATG
Kv4.3	TTCTTCTTCAATGAGGACACGA	CGGTAGAAGTTAAGCACACAAC
Kv5.1	CTGGGTGTTGACGCTGCTGAG	TGACCACGGAGGAGACAAGGATG
U6	GCTTCGGCAGCACATATACTAAAAT	CGCTTCACGAATTTGCGTGTCAT
mmu-miR-29b-2-5p	GCGCTGGTTTCACATGGTG	AGTGCAGGGTCCGAGGTATT

**Table 3:** Clinical characteristics of the subjects.

	Nonpregnancy (*n* = 135)	Pregnancy (*n* = 336)	Pregnancy+P4 (*n* = 117)
Ag (years)	31.49 ± 0.40	30.84 ± 0.23	30.81 ± 0.37
BMI (kg/m^2^)	24.33 ± 0.64	27.97 ± 0.38^∗∗^	28.22 ± 0.58
Gestational week	—	38.73 ± 0.08	38.9 ± 0.10
Gravidity	—	2.14 ± 0.07	2.14 ± 0.13
Parity	—	0.41 ± 0.03	0.43 ± 0.06
HR (bpm)	82.93 ± 1.01	92.88 ± 1.01^∗∗^	90.51 ± 1.21
QT (ms)	351.19 ± 2.48	341.56 ± 1.34^∗∗^	342.56 ± 2.30
QTc(ms)	409.91 ± 1.60	421.60 ± 0.97^∗∗^	417.81 ± 1.51^#^

P4: progesterone; HR: heart rate; QTc: heart-rate corrected QT (QTc) interval intervals calculated using Bazett's formula (QTc = QT/√RR). ^∗∗^*p* < 0.01 vs. nonpregnancy and ^#^*p* < 0.05 vs. pregnancy.

## Data Availability

The data in support of the results are available from the corresponding author on reasonable request.
